# Tfh1 Cells in Germinal Centers During Chronic HIV/SIV Infection

**DOI:** 10.3389/fimmu.2018.01272

**Published:** 2018-06-06

**Authors:** Vijayakumar Velu, Geetha Mylvaganam, Chris Ibegbu, Rama Rao Amara

**Affiliations:** ^1^Emory Vaccine Center, Emory University, Atlanta, GA, United States; ^2^Yerkes National Primate Research Center, Emory University, Atlanta, GA, United States; ^3^Ragon Institute of Massachusetts General Hospital, Massachusetts Institute of Technology, Harvard, Cambridge, MA, United States

**Keywords:** Tfh1 cells, germinal centers, HIV/SIV reservoirs, follicular CD8 T cells, Tfh cells, HIV/SIV infection

## Abstract

T follicular helper CD4 cells (Tfh) are essential for the development and maintenance of germinal center (GC) reactions, a critical process that promotes the generation of long-lived high affinity humoral immunity. It is becoming increasingly evident that GC-Tfh cells are heterogeneous in nature with some cellular characteristics associated with a Th1, Th2, and Th17 phenotype. Emerging studies suggest that GC-Tfh cells are directed to differentiate into distinct phenotypes during chronic HIV/SIV infection and these changes in GC-Tfh cells can greatly impact the B cell response and subclass of antibodies generated. Studies in HIV-infected humans have shown that certain Tfh phenotypes are associated with the generation of broadly neutralizing antibody responses. Moreover, the susceptibility of particular GC-Tfh subsets to HIV infection within the secondary lymphoid sites can also impact GC-Tfh/B cell interactions. In this review, we discuss the recent advances that show Tfh heterogeneity during chronic HIV/SIV infection. In particular, we will discuss the dynamics of GC-Tfh cells, their altered differentiation state and function, and their impact on B cell responses during HIV/SIV infection. In addition, we will also discuss the potential role of a recently described novel subset of follicular homing CXCR5^+^ CD8 T cells (Tfc) and their importance in contributing to control of chronic HIV/SIV infection. A better understanding of the mechanistic role of follicular homing CD4 and CD8 T cells during HIV/SIV infection will aid in the design of vaccines and therapeutic strategies to prevent and treat HIV/AIDS.

## Introduction

**Figure 1:**
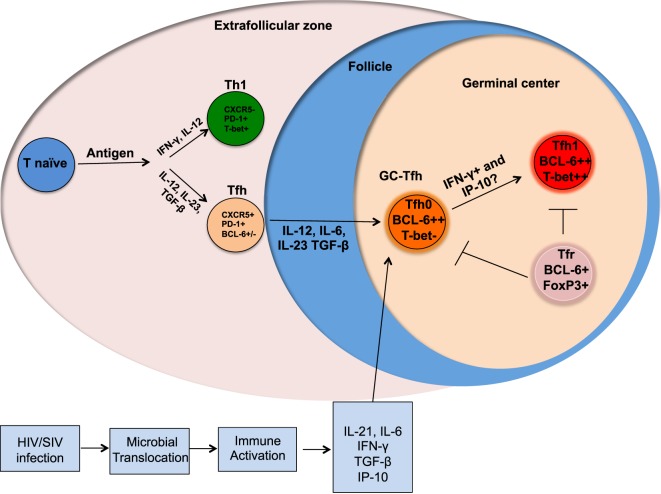
Altered differentiation of Tfh cells during chronic HIV/SIV infection. Following antigenic stimulation naïve CD4 T cells differentiate into different helper T cells and the presence of cytokines, such as IL-12, IL-23, and TGFβ promote differentiation into Tfh cells. Upon further interaction with B cells, these Tfh differentiate into germinal center (GC)-Tfh and migrate to GC. GC-Tfh can further differentiate into Tfh1 cells that can be mediated by the high levels of IFNγ and IP-10 produced during chronic HIV/SIV infection. The GC-resident Tfr cells can regulate the magnitude and function of GC-Tfh.

Lymphoid organs are the primary anatomical compartments for the generation of an effective adaptive immune response. CD4 T cells play a central role in the generation of adaptive immunity by providing help to both B cells and CD8 T cells (1, 2). CD4 T helper cells comprise of multiple subsets, including Th1, Th2, Th17, Tfh, Th9, Th22, Th-CTL, and T-regulatory cells (1, 3–5), and the generation of each subset is regulated by specific transcription factors and cognate cytokines (3). Among the various subsets of CD4 T cells, the follicular CD4 T cells (Tfh) reside in the B cell follicles and germinal centers (GC) of lymphoid tissue and play a major role in providing B cell help for the generation of high affinity antibody and long-lived memory B cell response (6, 7). Tfh cells are characterized by the expression of the chemokine receptor CXCR5 (required for homing to B cell follicles), PD-1, CD40L, and ICOS, and the transcription factor Bcl-6 (Figure 1) (8). These cells secrete the cytokines IL-21, IL-4, and IL-10 (6). A subset of Tfh cells reside within the GCs (GC-Tfh) (Figure 1), interact with GC-B cells, and facilitate affinity maturation and Ig class switching (9–12). The GC-Tfh cells express higher levels of PD-1 and Bcl-6 compared to Tfh cells that reside outside the GC. The interaction between Tfh and B cells is mediated by cell associated and soluble factors, including CD40L and ICOS, and IL-21, IL-10, and IL-4 (1, 6). GCs also consist of a subset of regulatory CD4 T cells called follicular regulatory cells (Tfr), which aid in regulating Tfh responses during GC reactions (Figure 1) (13, 14). Blood counterparts of lymph node (LN) resident Tfh have also been identified (15) and similar to LN-Tfh cells, these peripheral Tfh cells (pTfh) have been shown to provide help to B cells *in vitro* (15–17).

The linear multistage Tfh differentiation pathway implicates cooperation between multiple antigen-specific interactions and signaling pathways to imprint Tfh differentiation program in the secondary lymphoid organs (7). These include TCR activation, costimulation, cytokines and chemokine receptors. Now it is well established that the co-stimulatory receptors, such as ICOS, CD40L, and cytokines, such as IL-12, IL-23, TGF-β, IL-6, and SLAM family receptors regulate the Tfh differentiation program. Although IL-12 has been shown to be essential for Th1 differentiation, it has also been shown to be important for Tfh cell differentiation in humans (6, 17–20). An early step in the differentiation of human Tfh cells is the upregulation of CXCR5 that is strongly induced by the combination of cytokines IL-12, IL-23, and TGF-β (Figure 1) (18). The expression of cell surface CXCR5 allows for trafficking of Tfh cells along a CXCL13 chemokine gradient into lymphoid B cell follicles (21, 22). Recently, Activin A has been identified as a novel regulator that enhances the expression of multiple genes associated with the Tfh program (23), however, this program was conserved in humans and macaques but not in mice.

Tfh cells have been extensively studied in the LN of chronic HIV-infected humans and SIV-infected rhesus macaques (RM) (24–26). HIV infection is associated with altered T and B cell differentiation and enhanced frequencies of Tfh and B cell follicles within secondary lymphoid sites. Characterization of LN Tfh cells during chronic HIV infection has demonstrated impaired B cell help *in vitro* (27, 28). Furthermore, LN-resident Tfh cells are targeted early after SIV infection and constitute a major fraction of latent reservoirs during highly active anti-retroviral therapy (ART) (29–31). Despite their high susceptibility to HIV/SIV infection, many studies including our own reported an accumulation of both tissue resident (32, 33) and circulating Tfh cells during the early chronic phase of HIV/SIV infection (34, 35). In this review, we focus on the recent reports that studied the Tfh cell accumulation, differentiation and heterogeneity during chronic HIV/SIV infection, and discuss the influence of these changes in Tfh cells on the GC response.

## Dynamics of Tfh Cells during Chronic HIV and SIV Infections

**Figure 2:**
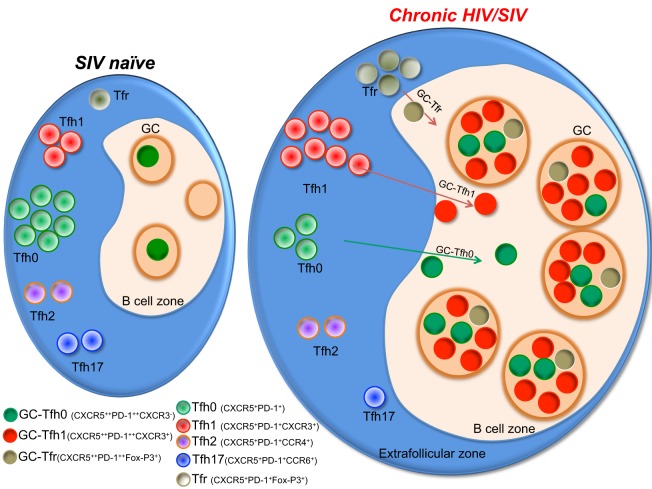
A model showing the status of different germinal center (GC)-Tfh cell subsets during chronic HIV/SIV infection. Tfh cells present as phenotypically distinct subsets with expression of different chemokine receptors pertaining to different lineages of helper CD4 T cells. Upon activation during chronic SIV/HIV infection, there is a massive enrichment of CXCR3^+^ Tfh cells within the Tfh population in the GC of the lymph node, which in turn drives rapid accumulation of Tfh1 cells. Typical progressors will present with hyperplastic follicles containing a high density of GC-Tfh cells with high levels of CXCR3 expression.

Multiple studies including our own have characterized the Tfh cells in the LNs during chronic HIV infection in humans (27, 29, 36, 37) and SIV infection in RMs (33, 35, 38–40). These studies demonstrated a marked increase in Tfh cells during chronic SIV infection and this increase in Tfh cells has been shown to be associated with higher HIV/SIV replication (27, 29, 33, 35, 38). Importantly, this increase in Tfh cells occurs despite their high frequency of infection *in vivo* and *in vitro*. Additionally, Tfh cells constitute a significant portion of the HIV/SIV reservoir (31, 32, 35, 36, 40–42). It has been shown that infection of Tfh cells occurs early in the course of SIV infection and persists throughout the course of disease progression (42). Although longitudinal studies in HIV-infected humans are yet to be done, cross-sectional studies suggest a similar profile of infection (27, 29, 31). However, Tfh in lymphoid tissues are eventually depleted in macaques with end-stage AIDS (40). It is also important to note that rapid progressing SIV infection results in severe follicular involution in lymphoid tissues, while on the contrary, animals that are typical progressors show lymphadenopathy with confluent GCs and follicular hyperplasia (43) (Figure 2). In addition, there is increasing evidence to suggest that follicular hyperplasia does not completely resolve following ART (44) and that the preferential carriage of HIV in Tfh cells during ART therapy (31, 45) contributes to the persistent and intractable viral reservoir in ART-treated patients. The mechanisms that contribute to increased Tfh cells during acute HIV/SIV infection are not completely clear. However, the increased levels of IL-6, TGF-β, and IL-21 during chronic HIV/SIV infection could contribute significantly to their expansion (Figure 1). In addition, factors such as relative exclusion of follicular CD8 T cells in B cell follicles, lack of regulation by Tfr (T follicular regulatory cells), lack of follicular NK cells, persistence antigen stimulation, and immune inflammation all these above factors contribute to the rapid accumulation of Tfh cells in lymphoid follicles during chronic HIV/SIV infection. Although there is an aberrant expansion of Tfh cells during chronic HIV infection, these cells are providing inadequate help to B cells. One important issue with GC-Tfh cell is the identification of antigen-specific GC-Tfh cells in lymphoid tissues. It is problematic to identify antigen-specific GC-Tfh cells by cytokine production, as GC-Tfh cells have been shown to be a poor cytokine producers compared to other memory CD4 T cell subsets, as biological role of GC-Tfh cell is to provide B cell help. In order to overcome this problem, currently investigators have started focusing on the cytokine-independent activation induced marker methodology assay (AIM assay using OX40 and CD25) to identify antigen-specific GC-Tfh cells in lymphoid tissues in human and macaques (46, 47). This AIM assay could identify (>10-fold) more antigen-specific GC-Tfh cells compared to cytokine assay. In addition, it has been shown that GC-B cells express higher levels of programmed cell death 1 ligand (PD-L1) in LN during chronic HIV infection, which may suggest a potential role for PD-1/PD-L1 interaction in regulating Tfh cell function (27). Moreover, engagement of PD-1 on Tfh cells leads to a reduction of Tfh cell proliferation, activation, cytokine production, and importantly ICOS expression (48), a key molecule in maintaining a Tfh phenotype. All together these data suggest that impaired Tfh-mediated B cell help diminishes B cell responses during HIV infection and may be regulated by the PD-1 axis on Tfh cells.

## Peripheral Tfh versus GC Tfh Cells

A subset of CD4 T cells in the blood expresses CXCR5 and is referred to as peripheral (pTfh) or circulating Tfh cells (17, 49, 50). These pTfh cells have been identified in mouse, macaques, and humans, and are considered to be the functional equivalent of Tfh cells in the LN. These pTfh cells express CCR7, albeit at low levels indicating that these cells traffic through lymphoid tissue. It has been shown that the pTfh cells express CXCR5 and PD-1 stably (15). However, pTfh express significantly lower levels of PD-1 and do not express Bcl-6 compared to GC-Tfh cells. Similar to LN Tfh, pTfh can also express other chemokine receptors associated with Th1 (CXCR3), Th2 (CCR4), and Th17 (CCR6) cells (17). pTfh can be distinguished into four subsets based on the expression of CXCR3 and PD-1. In humans, the PD-1^lo^ CXCR3^−^ pTfh express high levels of IL-4, do not express IFN-γ, and provide superior B cell helper function compared to PD-1^+^ CXCR3^+^ cells (15, 51). In addition, the presence of higher frequency of PD-1^lo^ CXCR3^−^ pTfh was shown to be associated with the development of a broader neutralizing antibody response in HIV-infected individuals with high viremia (15, 51). However, in another study the ratio of PD-1^lo^ to PD-1^hi^ cells within the CXCR3^+^ pTfh was shown to correlate with increased neutralization breadth in HIV controllers with very low viremia (34). These results suggest that both CXCR3^−^ and CXCR3^+^ pTfh expressing lower levels of PD-1 may be important for the generation of a functional antibody response. More studies in different disease contexts are required to correlate the phenotypic and functional differences previously observed between pTfh and GC-Tfh in order to understand the important dynamics of this subset in blood and tissue.

## HIV and SIV Infections Alter Tfh Differentiation toward Tfh1 Phenotype

The blood memory Tfh cells have been shown to co-express chemokine receptors associated with other T helper cell lineages, such as CXCR3 (Th1), CCR4 (Th2), and CCR6 (Th17) (17, 34, 52). Recent studies characterizing Tfh cells during chronic HIV/SIV infection have delineated phenotypically distinct subsets of Tfh cells in the circulation (17, 34, 53). Similarly, data from our recent study in macaques revealed that a significant proportion of GC-Tfh cells express the surface markers associated with several CD4 lineages during chronic SIV infection (Figure 2). We observed a selective enhancement of CXCR3^+^ Tfh (Tfh1) cells in the blood and LN and rapid depletion of CCR6^+^ Tfh cells (Tfh17) (32) demonstrating that SIV infection alters the balance of different subsets of Tfh cells. Others and we have also observed a marked enhancement of T-bet (Th1 marker) expression on Tfh and GC-Tfh cells (32, 33, 40, 54, 55). Unlike conventional GC-Tfh0 cells (CXCR3^−^), these GC Tfh1 cells exclusively produced IFN-γ (32). Interestingly, these T-bet^+^ Tfh1 cells also expressed Tfh transcription factor Bcl-6 (32, 54). The immune mechanisms that contribute to induction of Tfh1 cells are not completely understood. A similar Tfh1 phenotype was observed in GC-Tfh of humanized mice infected with HIV (56), during chronic LCMV clone-13 infection (57) and malarial infection (49). On the other hand, this phenotype may not be specific to chronic infections as we recently observed this phenotype on GC-Tfh cells after DNA/MVA SIV vaccination in RM (58). This raises the possibility that the local inflammation alone can induce this CXCR3 phenotype on GC-Tfh cells. One possibility is that the induction of high levels of CXCL10 (IP-10) during chronic infection and after DNA/MVA vaccination may promote induction of CXCR3 on Tfh cells (59).

The Tfh1 cells differ significantly compared to Tfh0 cells in terms of the expression of key molecules. Tfh1 cells, compared to Tfh0 cells express relatively lower levels (MFI) of CXCR5, PD-1, and CCR7 and higher levels of ICOS and IL-21 (Table 1). In addition, they exclusively produce IFN-γ express high levels of HIV co-receptor CCR5 and HIV-binding protein α4β7 suggesting that these cells are highly susceptible to HIV/SIV infection. Importantly, these cells also provide help to B cells similar to Tfh0 cells. One possible reason for better B cell help from Tfh1 cells could be the expression of high levels of ICOS, IL-21, and CD40L, as these markers direct recruitment of B cells and more efficiently activate B cells (60).

**Table 1:** Divergent marker profile of Tfh1 cells versus Tfh0 cells.

Markers	CXCR3^**+**^ Tfh	CXCR3^**−**^ Tfh
**Surface markers**		
CXCR5	+++	+++
CXCR3	+++	−
PD-1	++	+++
ICOS	+++	+++
CCR7	++	+/−
CCR5	+++	+/−
α4β7	+++	+/−
**Transcription factors**		
BCL-6	+++	+++
T-bet	+++	−
**Cytokines**		
IFN-γ	+++	−
IL-21	+++	+++
CD40L	+++	+++

## Tfr Cells during Chronic HIV/SIV Infection

Foxp3^+^ regulatory cells are important in suppressing different types of immune responses help to maintain homeostasis. Recent data in mice, macaques, and humans have identified a subset of Foxp3^+^ cells that express Tfh markers, such as CXCR5, PD-1, ICOS, and BCL-6, and migrate into the B cell follicles to regulate Tfh and B cell differentiation. These cells are called Tfr (follicular regulatory) cells (13, 14, 61). Although these cells show characteristics of Tfh cells they lack expression of functional molecules required for B-cell help, such as CD40L, IL-4, and IL-21. Deletion of Tfr cells or impairing their follicular localization led to increased number of Tfh and GC B cells in murine models (13, 14, 61). These cells inhibit GC reactions by interacting with Tfh cells. The mechanisms by which Tfr cells limit Tfh cell function are not clearly understood. During chronic HIV/SIV infection, Tfr cells are also expanded in parallel with Tfh cells. Although both Tfh and Tfr expand after SIVmac251 infection (62), higher frequency of Tfr cells is associated with lower Tfh frequency, suggesting that the expansion of Tfr cells diminishes Tfh frequencies and eventually B cell responses and antibody production (55, 63, 64). In line with this, a recent study demonstrated that a deficiency in Tfr cells promotes autoimmunity (65). *In vitro*, Tfr inhibit the ability of Tfh to proliferate and produce critical B cell helper cytokines, such as IL-4 and IL-21, although they maintain ICOS expression. A small fraction of HIV-infected individuals develops broadly neutralizing antibodies and it would be interesting to assess whether these individuals have lower frequencies of Tfr that would aid in the number and quality of Tfh and the generation of NAbs. Given their negative influence on Tfh cells, HIV vaccination modalities that induce lower levels of Tfr may generate stronger Tfh responses and higher quality B cell responses. Further studies are required to elucidate the role of Tfr on humoral immunity both post vaccination and during HIV/SIV infection.

## Tfh and HIV/SIV Viral Reservoirs

SIV infection of Tfh occurs early during primary infection and persists over the course of the disease (42). Although extensive longitudinal studies have not been carried out in humans, cross-sectional studies suggest a similar temporal profile of HIV infection of Tfh (29, 37). Studies have shown that HIV-specific CD4^+^ T cells are preferentially infected by HIV/SIV (66, 67). Within the CD4 compartment, the Tfh population has been shown to constitute higher numbers of HIV-infected cells that are more efficient in supporting viral replication and correlate directly with plasma viral RNA levels (36). Despite representing a large fraction of the HIV-infected CD4 pool (29), LN-resident Tfh cells appear to express low levels of the HIV co-receptor CCR5 (27, 29, 32, 33) but do, however, express CXCR4 (40). A recent study suggested that approximately 30% of human Tfh cells may be CCR5^+^ cells (41). Furthermore, studies have also shown that the proviral DNA sequences in Tfh cells from SIV-infected macaques are predominantly CCR5-tropic (42). The mechanism by which CCR5 tropic HIV/SIV is present at high levels in Tfh cells in macaques is not well understood. This leads to several questions; where and when are Tfh cells becoming infected, are they being infected before migrating into the GC at the stage of Tfh precursors which express high levels of CCR5 (32, 33), a potential mechanism recently described (40). In support of this potential mechanism is the knowledge that Th1 like Tfh cells accumulate during SIV infection, constitute a large proportion of the Tfh subset, and express higher levels of CCR5 and the HIV binding gut homing integrin receptor α4β7 (32). The accumulation of Tfh1 cells throughout the course of SIV infection both in periphery and in LN (32) suggest that Tfh1 cells accumulate rapidly post HIV/SIV infection and could potentially be transdifferentiating either from a different CD4 T helper cell subset or from a precursor population into a committed GC-Tfh phenotype. Several studies have reported the presence of CXCR3^+^CXCR5^+^ pTfh cells in HIV-infected individuals and in humanized mice preferentially expressing the HIV-co-receptor CCR5 (56, 68). Therefore, we can speculate that Tfh1 cells that are CCR5^+^α4β7^+^ may have the capacity to maintain a dynamic viral reservoir in GCs.

## Follicular Homing CD8 during HIV/SIV Infection

As described above, HIV and SIV infection of Tfh occurs very early in the course of infection and persists throughout disease even after ART (29, 42). Similarly, a higher fraction of these Tfh cells is shown to contain replication competent HIV genomes during ART (29, 36). An important question that needs to be addressed is the role of HIV-specific CD8 T cells in targeting and clearing the viral reservoirs that reside within Tfh cells. It is very clear that anti-viral CD8 T cells are critical for HIV/SIV control (69–74) even under ART (75). However, B cell follicles/GCs are considered to be immune privileged for anti-viral CD8 T cells (76, 77). Studies in unvaccinated SIVmac251-infected RM and HIV-infected humans showed that anti-viral CD8 T cells have a limited capacity to migrate to B cell follicles and GC of the lymphoid tissue during chronic infection (76–78) and the exclusion of CD8 T cells from GC sites have been posited as an important mechanism of immune evasion by HIV/SIV. However, recent studies reported the emergence of CD8 T cells expressing the chemokine receptor CXCR5 that is required for homing to B cell follicles (2, 10) during chronic LCMV, EBV, and HIV infections (79–82). Similarly, in the setting of DNA/MVA vaccinated and SIVmac251-infected macaques, we showed a rapid expansion of a novel subset of SIV-specific CD8 T cells expressing CXCR5 (Tfc) in vaccinated controllers after SIV infection (83) and the expansion of these cells was strongly associated with improved control of SIV replication. The higher expansion of these cells correlated strongly with the higher presence of anti-viral CD8 T cells in the GCs. Similar to SIV studies, these cells are shown to be present in the B cell follicles of HIV-infected LN (84). A more recent study carried out a comprehensive analysis of the phenotype, frequency, localization, and function of follicular CD8 T cells in SIVmac251-infected macaques (85). This study demonstrated that follicular CD8 T cell accumulation occurs in pathogenic SIV infection but not in natural infection (African green monkeys). Interestingly, this study provides clues to the cause of mobilization and accumulation of follicular CD8 T cells during pathogenic SIV infection and describes the process to be largely driven by inflammation and immune activation in and around B cell follicles. Similar to CD8 T cells, a recent study has shown that NK cells also traffic to the GCs, but these cells were seen only in non-pathogenic African green monkeys but these cells are rare SIV mac infection (86). These results clearly demonstrated that anti-viral CD8 T cells can migrate to B cell follicles under conditions of controlled SIV infection.

**Figure 3:**
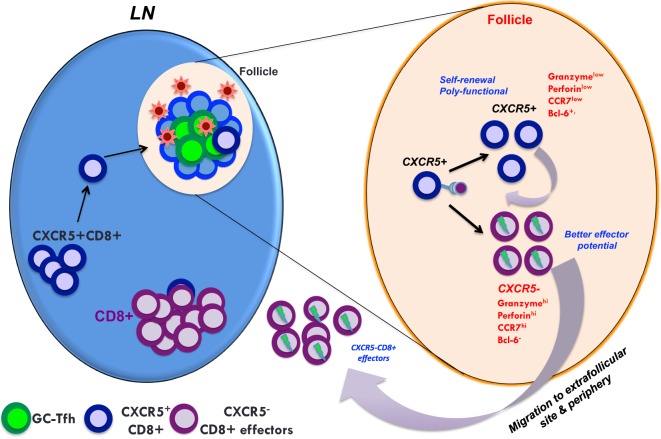
A model showing the predicted role of follicular homing CXCR5^+^ CD8 T cells in controlled SIV infection. In controlled SIV infection, the population of virus-specific CD8^+^ T cells expressing CXCR5 increases in lymphoid follicles. These CXCR5^+^ CD8 T cells can give rise to both CXCR5^+^ and CXCR5^−^ CD8 T cells that may be important for the maintenance of anti-viral CD8 T cells in both follicular and extrafollicular compartments. Thus, generating anti-viral CXCR5^+^ CD8 T cells by vaccination and therapeutic interventions may be critical for the control of chronic HIV/SIV infection.

The mechanisms by which these Tfc contribute to control of HIV/SIV are still under active investigation. Interestingly, Tfc possess a unique gene expression profile that shares both a cytotoxic CD8 T cells and Tfh phenotype (81, 83). These cells display enhanced poly functionality and are capable of restricting the expansion of SIV antigen-pulsed Tfh *in vitro* (83, 84). The Tfc from LN of HIV-infected individuals have been shown to possess higher cytolytic activity than extrafollicular CD8 T cells (79, 80, 82). Similar to HIV/SIV infection, CXCR5^+^ CD8 T cells have been identified during chronic LCMV infection. These cells have been shown to possess stem-cell like properties with self-renewal potential and may prove critical for long-term maintenance of effector CD8 T cells (Figure 3) (81). Thus, CXCR5^+^ CD8 T cells may contribute to viral control by replenishing the effector CD8 T cell population required to eliminate persistent virus. It is also important to note that some studies also suggest that the CD8 T cells that localize within B cell follicles may have limited cytolytic capacity and the overall frequencies of virus-specific CD8 T cells are lower in absolute numbers in the intra-follicular compared to extra-follicular compartment (78, 87, 88). However, the potential of Tfc to generate a population of effector CD8 T cells suggests that these cells may also contribute to viral control indirectly through their ability to homeostatically reconstitute the effector CD8 T cell response.

## Conclusion

The past few years of active research have provided profound insight into the role of follicular CD4 T cells in HIV pathogenesis. It is now well established that the Tfh population represents a major fraction of the viral reservoir and it is essential to develop HIV cure approaches capable of targeting and eliminating these cells. It is also clear that follicular homing CD8 T cells may serve as an important subset in targeting the Tfh reservoir. However, we need to develop a greater understanding of the mechanisms that contribute to the development and maintenance of viral reservoirs in Tfh cells under ART to design strategies to purge virus from this cellular site. The discovery of phenotypically distinct subsets of circulating Tfh cells in HIV infection and the potential for memory recall of Tfh cells in mice warrants further investigation into follicular CD4 T cells in an effort to inform vaccination strategies for HIV. Moreover, the heterogeneous nature of GC-Tfh and circulating Tfh cells can be harnessed for the generation of optimal vaccine-induced HIV-specific B-cell responses. A significant amount of work remains to uncover molecular signals that regulate the generation of follicular CD8 T cells and if this subset can be achieved by vaccination. In this regard, our ongoing work in nonhuman primates has shown that a CD40L adjuvanted DNA/MVA vaccine can strongly promote the generation of Tfc. Finally, further studies are needed to determine optimal strategies to utilize circulating and GC-Tfh cells during prime and boost immunization strategies to promote robust protective and long-lived antibody responses against HIV infection without promoting increased target cell susceptibility to HIV.

## Author Contributions

VV, RA wrote the review. VV, GM and RA designed the figures and the table. VV, CI and RR edited the text. VV composed and oversaw the chapters. All authors listed have made a substantial, direct, and intellectual contribution to the work and approved it for publication.

## Conflict of Interest Statement

The authors declare that the research was conducted in the absence of any commercial or financial relationships that could be construed as a potential conflict of interest.
